# Politicultural Linking: Inferences Between Political and Apolitical Traits

**DOI:** 10.1093/poq/nfaf047

**Published:** 2025-11-01

**Authors:** Gaetano Scaduto

**Affiliations:** PhD Candidate, Department of Sociology and Social Research, University of Milan Bicocca, Milan, Italy

## Abstract

Evidence concerning inferences between political and apolitical traits has grown exponentially in recent years. This thriving literature—dealing with research on political stereotypes and projection around sociodemographic, psychological, and lifestyle traits—is disconnected and needs to be placed under a unifying framework. To achieve this, we introduce “Politicultural Linking,” a concept subsuming political inferences from apolitical cues and apolitical inferences from political cues. Through an extensive literature review of the works produced since 2009, we discuss and classify research on this topic, identifying common features, strengths, and weaknesses, and depicting a comprehensive conceptual framework. Moreover, we identify relevant gaps in the literature: the underexploration of inferences involving lifestyle preferences, the overrepresentation of US-based studies, the overlooked role of projection, and the lack of non-survey-based research. Consequently, we aim to set the agenda for future studies on this topic.

## Introduction

References to “angry white men” conservatives (Kimmel 2015), “posh boy Tories” (Arnett 2013), or “latte-drinking, sushi-eating, Volvo-driving” liberals (Cursed Political Campaign Ads 2019) can be heard in political conversations among friends, satirical news shows, political advertisements, and politicians’ speeches. Although often lightheartedly spoken, these expressions signal the perceived associations between political and apolitical traits, a relevant and understudied portion of how people conceive politics. Through these associations, people draw inferences about others’ political preferences from what they look like, how they behave, or what they wear, eat, or drive. Conversely, individuals can form expectations regarding others’ sociodemographic characteristics, personality, and lifestyle preferences simply from knowing their voting preferences. The media plays on these associations, structuring and reinforcing them, as exemplified by the *New York Times*’s quiz asking readers to guess someone’s presidential choice by looking inside their fridge (Keefe 2020). Political élites use them to display the congruency between their behaviors and their political positions, and they get punished when failing to do so, as with Usha Vance getting booed by the RNC crowd when she revealed that her husband—vice presidential candidate J. D. Vance—likes to cook Indian and vegetarian food (Martel 2024).

Studies on the topic can be divided between those observing inferences on others’ political preferences drawn from apolitical cues—such as guessing someone’s partisanship from their income, personality, or food tastes (Settle 2018; Deichert 2019; Lee 2021; Carlson and Settle 2022; Titelman 2023)—and those about inferences on apolitical traits from political cues—such as observing the sociodemographic, personality, and lifestyle traits associated with certain political groups (Clifford 2014; Goggin and Theodoridis 2017; Busby et al. 2021; Claassen et al. 2021; Samuels, Mello, and Zucco 2024). Some studies discuss these inferences through stereotyping—attributing beliefs about the characteristics and behaviors of members of certain groups to the target (Hilton and Von Hippel 1996)—while others do so through projection (counter-projection)—assuming that inferential targets possess the same (opposite) traits as oneself (Ames 2004; Denning and Hodges 2022).

**Figure 2.**
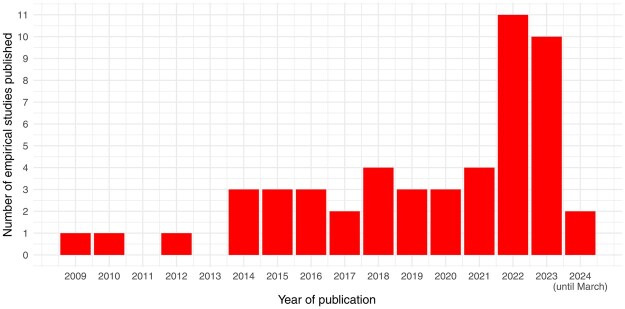
Studies concerning inferences between political and apolitical traits since 2009 (data from table 1).

While this stream of research has recently boomed (see figure 2), the existing literature is still disconnected and disorganized. This article proposes a solution to these problems by reviewing the evidence concerning inferences between political and apolitical traits in political science, political sociology, and political psychology. We identify the common features of these studies and introduce a comprehensive theoretical framework to discuss them. We bring order into a fragmented theoretical environment by presenting a novel higher-level unifying concept, subsuming political inferences from apolitical cues and apolitical inferences from political cues, and treating these as manifestations of the same underlying associations. We name this concept “Politicultural Linking” (PCL).^1^

The relevance of PCL lies in its potential to hinder cross-group contacts and foster polarization. PCL correlates with the same factors suggested to be linked with political polarization (e.g., news exposure, political interest; see section 4), and can directly foster political sectarianism (Finkel et al. 2020). Scholars have long argued that people inadvertently signal political views through traits like age, ethnicity, or clothing choices (MacKuen 1990). These signals are interpreted by people’s “detection system” (Carlson and Settle 2022) influencing decisions to engage in conversations (MacKuen 1990; Lee 2021; Carlson and Settle 2022; Scaduto and Negri 2024) and affecting what they feel, say, and learn during these (Balietti et al. 2021; Carlson and Settle 2022). Through PCL, individuals can quickly evaluate if someone is politically compatible and, consequently, whether they are willing to be friends, colleagues, or seat-neighbors with them (Deichert 2019; Lee 2021), preventing the spontaneous occurrence of cross-group contacts. Given that cross-group contacts are suggested to be an effective antidote to political polarization (Hartman et al. 2022; Levendusky and Stecula 2023; Rossiter 2023), an instrument preventing them further is a serious societal concern. Simultaneously, awareness about PCL might discourage individuals from adopting or displaying traits associated with the political outgroup (or not displaying the ones associated with the ingroup) for fear of being politically misperceived, fostering even more the alignment between political and apolitical traits (see section 2) and consequently reinforcing PCL in a self-fulfilling fashion.^2^

Moreover, through PCL people can infer others’ apolitical traits from awareness about their political positions, adjusting their behavior accordingly. Knowing others’ political preferences indeed affects the likelihood of hiring (Gift and Gift 2015; Lyons and Utych 2022) or working for someone (McConnell et al. 2018), as well as the selection of roommates (Shafranek 2021), coworkers (Baldassarri and De Jong 2025), neighbors (Theodoridis, Goggin, and Deichert 2023), and romantic/sexual partners (Huber and Malhotra 2017), again hindering contact between political groups.

On the other hand, PCL might also have positive consequences. For example, it may foster intragroup cohesion and trust (Lee 2021). Its link with polarization may lead to broader democratic participation and to excluding antidemocratic actors (Wagner 2024). Moreover, studies suggest that these associations may not be far from the actual correlations between political and apolitical traits (Carlson and Hill 2022; Orr and Huber 2022), making PCL a useful prediction tool.

The rest of the article is structured as follows. Section 2 details the conceptual framework surrounding PCL, discussing its novel contributions compared with previous conceptualizations, while section 3 presents a literature review of the related empirical evidence. Although our theoretical framework stems from the literature review, we suggest that readers familiarize themselves with the conceptual definitions introduced in section 2 before reading section 3. Section 4 sets the research agenda for future works on PCL, discussing current gaps in the literature—such as the underobserved role of projection, the methodological homogeneity of the studies, and the overlooked role of lifestyle-based inferences—and delving into relevant questions to be addressed by future studies. Finally, section 5 presents the conclusions.

## Toward a Definition of Politicultural Linking

*“Deciding what is and what is not political is a fraught, perhaps intractably opaque, matter”* (Bernstein 2018, p. 2).

We introduced PCL as a concept subsuming political inferences from apolitical traits and apolitical inferences from political traits.^3^ To the best of our knowledge, the academic community has not produced a single, unambiguous definition of what is “political.” Yet, to define PCL, we must first clearly state what we indicate as “political” and “apolitical” traits.

Given the links between PCL and the literature on stereotyping and projection (see section 3), grounded in the concept of social groups (Hilton and Von Hippel 1996; Ames 2004), we consider “political traits” those traits unambiguously signaling membership to a political group. For our purposes, political groups are groups characterized by a shared (positive or negative) ideology, partisanship, position on political issues, vote choice, or support for political candidates.^4^ Therefore, for example, “conservative,” “Democrat,” “pro-choice,” “Trump supporter,” or “antifascist,” “antipetista” (Samuels, Mello, and Zucco 2024), and “Never Trumper” are, according to this definition, political traits. Complementarily, with “apolitical traits” we indicate traits not satisfying our definition of “political,” such as gender, extraversion, or omnivorousness.

Certain lifestyle traits, possibly adopted for political reasons, may arguably be considered intrinsically political. For example, a person can be vegan for environmental reasons. Yet, these traits do not *unambiguously* signal political traits—one could be vegan for health-related reasons while being a climate-change denier. Thus, we consider them apolitical. Moreover, the motivations behind a certain lifestyle preference are unlikely to influence PCL. Indeed, information regarding these is usually not available in the early stages of social interactions when information about the counterpart’s political traits is missing and inferences are needed to fill the information gap.

Political and apolitical traits can be related both in reality and in perceptions. We define “the alignment between political and apolitical traits” (to shorten, “the alignment”) as a situation where political and apolitical traits show significant co-occurrence, ending up being correlated. For example, evidence of the alignment is found in studies observing that liberals more often belong to sexual minorities (Ahler and Sood 2018), are more open to new experiences (Vecchione et al. 2011), and are more interested in electric cars and basketball (Hetherington and Weiler 2018; Praet et al. 2022).

The alignment between sociodemographic and political traits has been an object of research since the early days of modern political science (Lipset and Rokkan 1967), and the alignment of psychological and political traits is among the main research topics in political psychology (Federico and Malka 2023). A recent stream of literature focused on the alignment between political and lifestyle preferences, addressing it with several different expressions, such as “lifestyle politics” (DellaPosta, Shi, and Macy 2015), “politicultural sorting” (Rogers 2022), “the oil-spill” (DellaPosta 2020; Rawlings and Childress 2024), and “lifestyle polarization” (Talaifar et al. 2024). Rogers and Jost (2022) recently pointed out ideological asymmetries in lifestyle alignments: while conservatives engage exclusively in their distinctive preferences, liberals behave as cultural omnivores, substantially engaging in distinctly conservative cultural activities as well.

We call “associations between political and apolitical traits” (to shorten, “associations”) the set of one’s perceived alignments. For example, perceiving atheism and Democratic partisanship as correlated (Orr and Huber 2022), or loyalty and opinion on the death penalty (Clifford 2014), or drinking lattes and liberalism (DellaPosta, Shi, and Macy 2015; Mutz and Rao 2018), are examples of associations.

The alignment may engender associations (Hilton and Von Hippel 1996; Goldberg and Stein 2018; Cerulo, Leschziner, and Shepherd 2021), but it is neither a necessary nor a sufficient condition (Hilton and Von Hippel 1996; Bordalo et al. 2016; Goldberg and Stein 2018; Ahler and Sood 2023). Associations may also arise from observing other co-occurrences, for example through media representations (Rahn and Cramer 1996), often employing exemplars (Bigsby, Bigman, and Martinez Gonzalez 2019; Myers 2020), or élite characteristics (Deichert 2019).^5^

Associations fuel inferential mechanisms selected after evaluating the perceived similarity with the target: when similarity is perceived, the subject might project, while perceived dissimilarity usually leads to stereotyping or counter-projection (Ames 2004; Denning and Hodges 2022).

Projection describes the process unfolding “when the perceiver assumes a target has the same mental states that he or she has or would have” (Ames 2005, p. 163). It concerns the idea that others are similar to oneself, that one’s opinion and co-occurrences are the most common, and that there is a “false consensus” around one’s preferences (Robbins and Krueger 2005). Complementarily, counter-projection, also called “negative projection” (Castelli, Arcuri, and Carraro 2009; Amira 2018), concerns the idea of seeing in others the opposite of oneself (Denning and Hodges 2022).

Inferences can also rely on stereotyping. Among the several conceptualizations, starting from Lippman’s (1921) “pictures in our heads,” stereotypes have been described as “associative networks of linked attributes” (Hilton and Von Hippel 1996, p. 240) around a target’s social category (Sened et al. 2025). What we call “associations” can therefore be conceived as the building blocks of the “associative network” around political traits.

**Figure 1.**
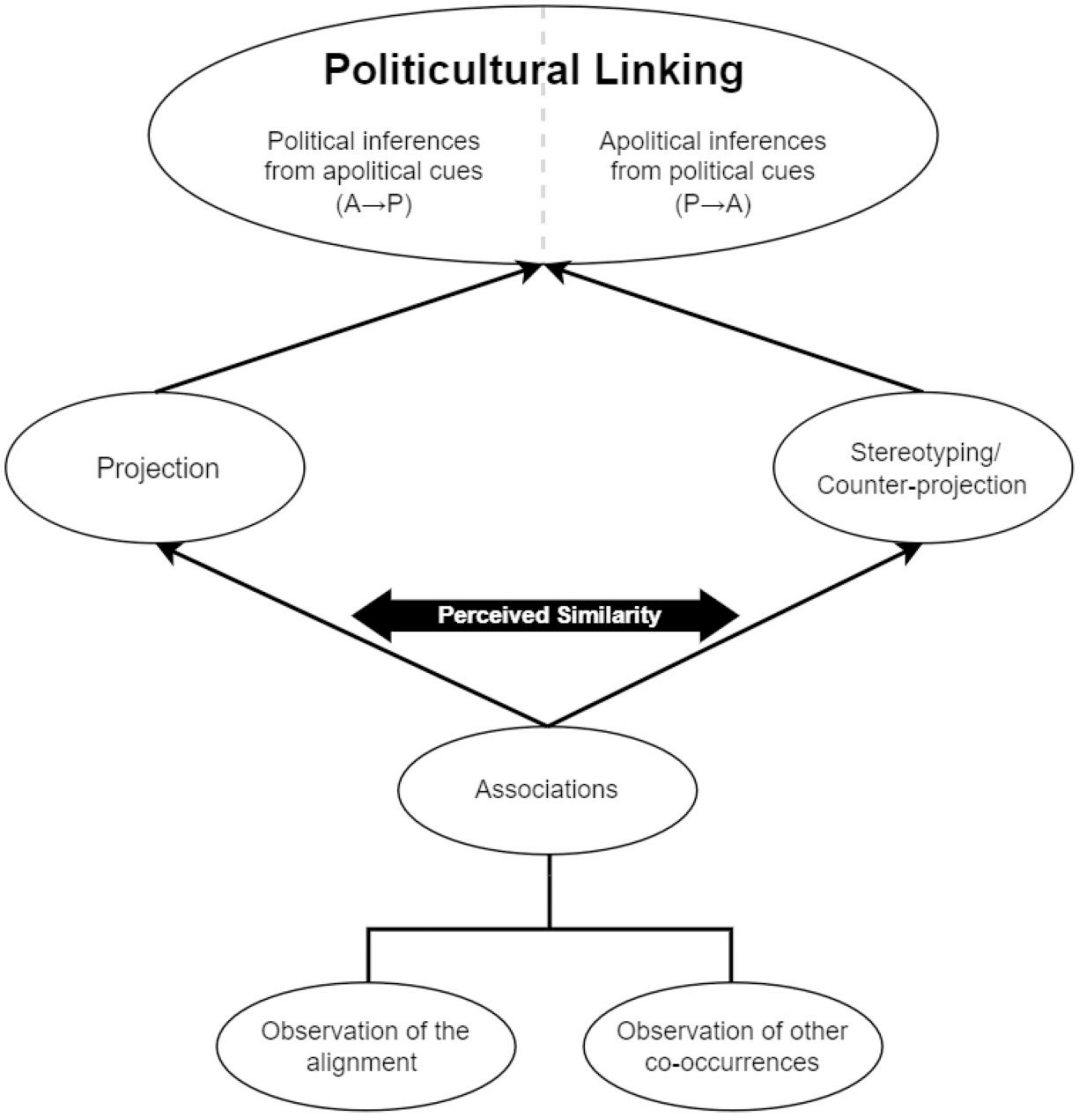
Representation of the conceptual framework around PCL.

Inferential strategies constitute the basis for “political inferences from apolitical cues” (A→P) and for “apolitical inferences from political cues” (P→A). Therefore, if leftism and Birkenstock shoes are associated, people may infer someone’s leftism from observing them wearing Birkenstocks (A→P). Conversely, they may infer that they own a pair of Birkenstocks from knowing they voted Green Party (P→A). To refer indistinctly to the two inferential directions, we introduce Politicultural Linking: the production of inferences about others’ political or apolitical characteristics based on the association between these. The conceptual framework is summarized in Figure 1.

As we show in section 3, PCL adds a series of features to the literature. First, it simultaneously accounts for both possible inferential directions, placing them under one conceptual unity as manifestations of underlying associations between political and apolitical traits. PCL therefore subsumes other unidirectional conceptualizations, such as the “detection system” (Carlson and Settle 2022), “perception of the composition of parties” (Claassen et al. 2021), and “political inferences from everyday cues” (Lee 2021), under a broader framework, allowing scholars to relate the conclusions from studies on different inferential directions and expanding the range of empirical strategies to investigate the causes and consequences of PCL. For example, through PCL it is possible to connect Carlson and Settle’s (2022) finding that the propensity to draw A→P is associated with one’s partisan strength with Ahler and Sood’s (2018) analogous finding regarding P→A.

Second, PCL explicitly places (political) inferences engendered by (counter-)projection and stereotyping under the broader framework of social inferential strategies, recognized by the psychological literature but often unaccounted for by social scientists (Ames 2004; Sened et al. 2025). This again helps in comparing similar and contrasting findings (Lerman and Sadin 2016; Van Oosten 2022) while also helping researchers and policymakers craft solutions to the consequences of PCL or exploit these to their advantage (see section 4). Moreover, since projection has been almost exclusively studied by psychological research, it also urges social scientists to account for this inferential strategy.

Third, while previous studies focused on “partisan stereotypes” (Busby et al. 2021; Ahler and Sood 2023), “ideological stereotypes” (Jones 2014), or “trait inferences from a politician issue stance” (Clifford 2014), PCL encompasses research dealing with a broader set of political traits, functioning as a more flexible conceptual tool, applicable to many different contexts and novel and extemporaneous political groups, such as Brexiters/Remainers, or vaxxers/anti-vaxxers.

Fourth, unlike Goggin and Theodoris’s (2017) “party reputations,” Mason and Wronski’s (2018) “subjective social sorting,” or Busby and colleagues’ “partisan stereotyping” (2021; see also Rothschild et al. 2019), PCL does not concern inferences between political traits (P→P). Therefore, it excludes from its scope inferences such as assuming a Republican is against environmental protection (Goggin, Henderson, and Theodoridis 2020). This distinction helps interpret the evidence suggesting inferences between political and apolitical traits and those between political traits have different causes and consequences (e.g., political knowledge is not related to the former but it is to the latter, while the opposite holds for affective polarization; see Rothschild et al. 2019).

Fifth, it explicitly accounts for inferences around lifestyle traits—neglected by most previously cited conceptualizations—therefore incorporating concepts such as “partisan cultural stereotypes” (Deichert 2019) and “everyday partisan stereotypes” (Lee 2021). This fills a gap in the perceptual equivalents of political correlates. The perceptual equivalent of the correlations between political traits (e.g., Baldassarri and Gelman 2008; Lelkes 2016) can be found in studies observing inferences on ideology/partisanship from issue positions (P→P) or vice versa (e.g., “associative issue ownership”; see Walgrave, Lefevere, and Tresch 2012). Busby and colleagues’ (2021) “coalitional” and “identity stereotypes” already conceptualized the perceptual equivalent of the correlations between sociodemographic/psychological traits and political preferences. However, the thriving literature regarding the politics-lifestyles alignment (DellaPosta, Shi, and Macy 2015; DellaPosta 2020; Rogers 2022; Rawlings and Childress 2024) lacks such an equivalent. When observed on lifestyle, PCL constitutes the perceptual equivalent of Talaifar and colleagues’ (2024) “lifestyle polarization,” Schoenmueller’s (2023) “preference polarization,” or DellaPosta and colleagues’ (2015) “Lifestyle Politics.”

## Reviewing the Empirical Evidence of Politicultural Linking

In this section, we review, discuss, and classify empirical studies researching either political inferences from apolitical cues (A→P) or apolitical inferences from political cues (P→A). We considered English-written works produced between 2009 and March 2024 without discipline limitation, allowing us to preserve the homogeneity of the literature reviewed while collecting a substantial number of studies. Although the timeframe selected limits the review’s comprehensiveness, we believe the conclusions drawn regarding the features, gaps, and future directions of this research stream are not substantially weakened by it, considering its recent expansion and the relative lack of studies published between 2009 and 2013 (see figure 2).

Given the conceptual heterogeneity of the studies reviewed, we employed a flexible semi-systematic literature review approach (Snyder 2019).^6^ The initial search was conducted using the search phrase: “*‘political stereotypes’ OR ‘partisan stereotypes’ OR ‘political projection’ OR ‘political inferences’ OR ‘politics and lifestyle perceptions’ OR ‘politics and trait perceptions’ OR ‘political inferences from apolitical cues’ OR ‘apolitical inferences from political cues’*” for studies published between 2009 and 2023. This search yielded 248 results on JSTOR, 76 on Web of Science, 135 on Scopus, and 4,180 on Google Scholar. We first inspected titles and abstracts,^7^ after which a full-text reading followed. We then integrated our collection retrieving works referenced in the most comprehensive recent studies on political stereotypes (Busby et al. 2021; Lee 2021; Ahler and Sood 2023; Carlson and Settle 2022; Hiaeshutter-Rice, Neuner, and Soroka 2023) and projection (Lerman and Sadin 2016; Denning and Hodges 2022; Kevins and Lee 2023), using the literature mapping software LitMaps,^8^ and following the anonymous reviewers’ suggestions.^9^ Moreover, we repeated the search including works published in the first three months of 2024. We also include works published in 2024 whose unpublished versions were retrieved before March 2024. This procedure yielded 53 studies that we classified into several categories to discuss the strengths and gaps in the literature. In what follows, all the percentages refer to table 1.

**Table 1.** Categorization of the empirical studies dealing with PCL.

Study	Found evidence?	Direction of the inference	Target of the inference	Political trait	Apolitical trait	Mentioned inferential strategy	National context	Empirical strategy
Ahler & Sood (2023)	Yes	A→P	People	Partisanship	Socdem. + Lifestyle	Stereotyping	US	Survey experiment
Ahler and Sood (2018)	Yes	P→A	People	Partisanship	Socdem.	Stereotyping	US	Survey experiment +cross-sect.
Amira (2018)	Partial	A→P	Politicians	Ideology	Psych.	Projection + Counter-projection	US	Survey experiment
Arnesen et al. (2019)	Yes	A→P	Politicians	Issues	Sociodem	None	NO	Survey experiment
Ash et al. (2020)	Yes	A→P	Politicians	Partisanship	Other	Stereotyping	US	Survey experiment
Barber and Pope (2022)	Partial	A→P	People	Partisanship	Socdem + Lifestyle	Stereotyping	US	Survey experiment
Bell et al. (2022)	Yes	P→A	People	Partisanship	Psych.	Projection	US	Survey cross-sect.
Bittner (2015)	Yes	P→A	Politicians	Ideology	Psych.	Stereotyping	AU + CA + GE + NZ + SW + UK + US	Survey cross-sect.
Bordalo et al. (2016)	Yes	P→A	People	Ideology	Psych.	Stereotyping	US	Survey cross-sect.
Bouchard (2022)	Yes	A→P	Politicians	Partisanship	Socdem.	Stereotyping	CA	Focus groups
Bruchmann et al. (2018)	Yes	P→A	People	Ideology + Partisanship	Psych.	Stereotyping	US	Survey experiment
Busby, Howat, and Daniel Myers (2024)	Yes	P→A	People	Partisanship	Socdem. + Psych.	Stereotyping	US	Survey cross-sect.
Busby et al. (2021)	Yes	P→A	People	Partisanship	Socdem. + Psych.	Stereotyping	US	Survey experiment + cross-sect.
Carlson and Hill (2022)	Yes	A→P	People	Other	Socdem.	Stereotyping	US	Survey experiment
Carlson and Settle (2022)	Yes	A→P	People	Ideology + Partisanship	Socdem. + Psych. +Lifestyle +Others	Stereotyping	US	Survey experiment
Casiraghi et al. (2024)	No	A→P	Politicians	Other	Socdem.	Stereotyping	IT	Survey experiment
Castelli et al. (2009)	Yes	P→A	Politicians	Other	Others	Projection + Counter-projection	IT	Lab experiment
Claassen et al. (2021)	Yes	P→A	People	Partisanship	Socdem.	Stereotyping	US	Survey experiment + Survey cross-sect.
Clifford (2014)	Yes	P→A	Politicians	Issues	Psych.	Stereotyping	US	Survey experiment + cross-sect. + Lab experiment
Clifford (2020)	Yes	P→A	People	Partisanship	Psych.	Stereotyping	US	Survey experiment
Clifford (2022)	Yes	P→A	Politicians	Issues	Psych.	Stereotyping	US	Survey experiment +cross-sect.
Coronel and Federmeier (2016)	Partial	P→A	People	Issues	Socdem.	Stereotyping	US	Lab experiment
Deichert (2019) ^a^	Yes	A→P	People + Politicians	Partisanship	Socdem. + Lifestyle	Stereotyping	US	Lab experiment + Survey experiment
Denning and Hodges (2022)	Yes	P→A	People	Other	Psych. +Lifestyle	Projection + Counter-projection	US	Survey experiment
Eriksson and Funcke (2015)	Yes	P→A	People	Partisanship	Psych.	Stereotyping	US	Survey
Goggin, Henderson, and Theodoridis (2020)	Partial	A→P	People	Ideology + Partisanship	Socdem.	Stereotyping	US	Survey experiment
Goggin and Theodoridis (2017)	Partial	P→A	Politicians	Partisanship	Psych.	Stereotyping	US	Survey experiment
Graham et al. (2012)	Yes	P→A	People	Ideology	Psych.	Stereotyping	US	Survey experiment
Hiaeshutter-Rice et al. (2023)	Yes	P→A, A→P	Politicians	Partisanship	Lifestyle	Stereotyping	US	Survey experiment
Jacobsmeier (2014)	Partial	A→P	Politicians	Ideology	Socdem.	Stereotyping	US	Survey cross-sect.
Jones (2014)	Yes	A→P	Politicians	Ideology + Partisanship + Issues	Socdem.	Stereotyping	US	Survey experiment
Jones and Brewer (2019)	Yes	A→P	Politicians	Ideology	Socdem.	Stereotyping	US	Survey experiment
Kane et al. (2021	Yes	A→P	People	Partisanship	Socdem.	Stereotyping	US	Survey cross-sect.
Kevins and Lee (2023)	Yes	A→P	Politicians	Partisanship	Socdem.	Projection + Counter-projection	CA + UK + US	Survey experiment
Lee (2021)	Yes	A→P	People	Partisanship	Socdem. + Lifestyle	Stereotyping	US	Survey experiment
Lerman and Sadin (2016)	Yes	A→P	Politicians	Ideology	Socdem.	Projection + Stereotyping	US	Survey experiment
Licata and Méon (2024)	Yes	A→P	Politicians	Ideology	Socdem. + Lifestyle	Stereotyping	FR	Survey cross-sect
López Ortega (2024)	Partial	A→P	Politicians	Partisanship	Socdem. + Psych.	Stereotyping	SP	Survey experiment
Myers (2023)	Partial	A→P	People	Partisanship	Socdem. + Psych.	Stereotyping	US	Survey experiment
Orr and Huber (2022)	Yes	P→A	People	Partisanship	Socdem.	Stereotyping	US	Survey experiment
Ouellet and Tremblay-Antoine (2024)	Yes	A→P	People	Partisanship	Socdem. + Lifestyle	Stereotyping	CA	Survey experiment
Rothschild et al. (2019)	Yes	P→A	People	Partisanship	Socdem. + Psych.	Stereotyping	US	Survey cross-sect.
Samuels et al. (2024)	Yes	P→A	People	Partisanship +Other	Socdem.	Stereotyping	BR	Survey cross-sect.
Scaduto and Negri (2024) ^a^	Yes	A→P	People	Ideology	Lifestyle	Projection + Stereotyping	IT	Survey experiment
Scheffer et al. (2022)	Yes	P→A	People	Partisanship	Psych.	Stereotyping	US	Survey cross-sect. + Field study
Scherer et al. (2015)	Yes	P→A	People	Partisanship	Psych.	Stereotyping	US	Survey cross-sect.
Settle (2018)	Yes	A→P	People	Ideology + Partisanship	Lifestyle	Stereotyping	US	Survey experiment
Titelman (2023)	Yes	A→P	People	Unclear	Socdem.	None	UK	Survey experiment
Titelman and Lauderdale (2023)	Yes	A→P	People	Other	Socdem.	None	UK	Survey experiment
Van Oosten (2022)	Yes	A→P	Politicians	Issues	Socdem.	Projection + Stereotyping	FR + GE + NL	Survey experiment
Van Trappen (2023)	Yes	A→P	Politicians	Ideology	Socdem.	Stereotyping	BE	Survey experiment
Visalvanich (2017)	No	A→P	Politicians	Ideology	Socdem.	Stereotyping	US	Survey experiment
Winter (2010)	Yes	A→P	Parties	Partisanship	Psych.	Stereotyping	US	Survey cross-sect.

a Unpublished work when conducting the literature review.

Most studies reviewed found significant and unambiguous evidence of PCL (81.1 percent). Although this result might be affected by publication bias (Wagner 2022), our review strongly suggests that PCL is an existing phenomenon in social reality. Some studies (15.1 percent) found limited evidence of PCL. In some cases, only a minority of the apolitical traits tested were significantly associated with political traits, as in Goggin and colleagues’ (2020) conjoint experiment where only four out of 28 apolitical attribute levels were significantly associated with perceiving someone as conservative. In other cases, the effects were detected only in part of the sample, showing that, for example, only white respondents inferred black politicians’ political traits from their race (Jacobsmeier 2014). Two studies find no significant evidence of PCL (Visalvanich 2017; Casiraghi, Curini, and Nai 2024).

As detailed in section 2, PCL subsumes two inferential directions: political inferences from apolitical cues (A→P) and apolitical inferences from political cues (P→A). Yet, these studies consider only one direction, either asking, for example, to list the apolitical traits associated with parties (43.4 percent) or to guess someone’s ideology after being informed of their apolitical characteristics (58.5 percent). The only partial exception is the study by Hiaeshutter-Rice and colleagues (2023), where the authors first asked respondents to name lifestyle items associated with political parties and then used these answers to create politicians’ pictures from which participants in another study were asked to infer partisanship.

Regarding inferential targets, research is clearly divided between studies concerning inferences targeted to politicians (39.6 percent) and to everyday partisans (58.5 percent),^10^ with one study considering parties as inferential targets (Winter 2010).

We identified four subcategories of apolitical traits considered in the studies. “Sociodemographic traits” (60.4 percent) concerns sociodemographic variables such as age, gender, and ethnicity. For example, studies looked at inferences based on race (Jacobsmeier 2014; Jones 2014), sex (Coronel and Federmeier 2016), and gender identity (Jones and Brewer 2019) and observed that liberals are perceived as less religious (Goggin, Henderson, and Theodoridis 2020), poorer, and more racially and sexually diverse (Ahler and Sood 2018) than conservatives.

“Psychological traits” (39.6 percent) refer to individuals’ personality, moral, or character traits (e.g., “femininity,” Winter 2010; “humbleness,” Amira 2018; “narrow-mindedness,” Carlson and Settle 2022; “laziness,” Myers 2023).^11^ A substantial substream of this literature used established lists of psychological traits, such as moral traits^12^ (Graham, Nosek, and Haidt 2012; Clifford 2014; Bordalo et al. 2016; Bruchmann, Koopmann-Holm, and Scherer 2018; Clifford 2020, 2022; Denning and Hodges 2022; Scheffer et al. 2022) or the “Big Five” personality traits (Denning and Hodges 2022; López Ortega 2024), as the apolitical end of the inference. Yet, most studies selected the traits to observe either from previous studies (e.g., Myers 2023), arbitrarily, or based on the availability of secondary data (e.g., ANES; see Goggin and Theodoridis 2017). A few studies observed psychological traits based on open answers describing members of a political group apolitically (Rothschild et al. 2019; Busby et al. 2021; Busby, Howat, and Daniel Myers 2024). When dealing with P→A, the studies observing these traits often refer to the concept of “trait ownership” (Hayes 2005).

Among other things, these studies observed the perceived associations between liberalism and compassion, femininity, and fairness, and between conservativism and loyalty, masculinity, and respect (Winter 2010; Graham, Nosek, and Haidt 2012; Scherer, Windschitl, and Graham 2015; Clifford 2020; Scheffer et al. 2022).

“Lifestyle traits” (20 percent) concern lifestyle and cultural preferences, such as fashion choices (Deichert 2019), food preferences (Carlson and Settle 2022; Scaduto and Negri 2024), favorite restaurants (Settle 2018), coffee shops (Lee 2021), cars (Lee 2021; Barber and Pope 2022), seasons, or pets (Denning and Hodges 2022). Studies highlighted how US liberals are perceived as latte-drinking ethnic-cuisine-enjoying electric-car drivers, and conservatives as black-coffee-drinking and cheeseburger-eating truck drivers (Settle 2018; Deichert 2019; Lee 2021; Hiaeshutter-Rice, Neuner, and Soroka 2023).

We also introduced a residual subcategory, “Other” (5.7 percent), containing three studies not fitting in the above subcategories: Carlson and Settle’s (2022) experiment where participants guessed people’s ideology from their names—Kurt and Duane were perceived as conservative, Liam and Dwayne as liberal—Castelli and colleagues’ (2009) study observing participants projecting their birthday on politicians, and Ash and colleagues’ (2020) study on partisan inferences from politicians’ accents.

Only a few studies compare the relative strength of inferences engendered by different categories of apolitical traits (Myers 2023, p. 641). Rothschild and colleagues (2019, p. 434) observe that psychological traits are mentioned twice as often as sociodemographics in open answers describing partisans’ apolitical traits (see also Busby et al. 2021; Busby, Howat, and Daniel Myers 2024). Myers (2023) finds that most psychological traits are statistically significant when inferring partisanship in a conjoint experiment, while most sociodemographics are not. When significance is reached, the magnitude of the effect is comparable: for example, being “atheist” (compared with “Catholic”) and being “lazy” (compared with “hardworking”) both significantly reduce the likelihood of perceiving someone as a Republican by 2–3 percentage points. Denning and Hodges (2022) found that people are likelier to project psychological than lifestyle traits to their political ingroup members, while they are more prone to counter-project lifestyle rather than psychological traits to the political outgroup. Regarding the relationship between lifestyle and sociodemographic traits, Deichert (2019) observes that veganism and liking yoga are more typically associated with Democrats than (respectively) being an atheist and being working class, while liking *Duck Dynasty* is considered more typical for a Republican than being evangelical.

Some studies observed partisan or ideological inferences from apolitical cues in a setting where explicit political information, such as issue positions, is also available (Goggin, Henderson, and Theodoridis 2020; Busby et al. 2021; Barber and Pope 2022; Casiraghi, Curini, and Nai 2024; López Ortega 2024; Myers 2023). These studies consistently show that the association between apolitical and political traits (partisanship, ideology) weakens or becomes nonsignificant in such settings, suggesting that, when available, political cues are preferred over apolitical cues to draw inferences on political traits (see Barber and Pope 2022 for a discussion).

Partisanship is the primary political trait employed to observe PCL (58.5 percent), followed by ideology (30.2 percent) and issue positions (11.3 percent). Studies also used traits such as support for presidential candidates (Denning and Hodges 2022), vote choices (Carlson and Hill 2022; Titelman and Lauderdale 2023), party coalition support (Castelli, Arcuri, and Carraro 2009), and populism (Casiraghi, Curini, and Nai 2024). Samuels and colleagues (2024) employed negative partisanship,^13^ while the study from Titelman (2023) asked respondents about their perceived “political commonality,” but did not indicate a specific trait through which this commonality should be observed.

With a few exceptions (Arnesen, Duell, and Johannesson 2019; Titelman 2023; Titelman and Lauderdale 2023), all the works reviewed mention more or less explicitly in their theoretical framework either projection, counter-projection, or stereotyping as the observed inferential strategy.

Studies relying on projection (15.1 percent) or counter-projection (7.5 percent) are less common and mostly published in social and political psychology journals such as *Political Psychology*, while those adopting a stereotype-based framework (84.9 percent) are more frequent in political sciences and often move from the theoretical underpinnings of Social Identity Theory (Tajfel and Turner 1979) applied to the political realm, according to which individuals develop social identities around (political) groups (Huddy 2001; Devine 2015) and use these social identities to categorize people accentuating perceived cross-group differences (Trepte and Loy 2017).

Observing which inferential strategy occurs is challenging. Yet, two studies (Lerman and Sadin 2016; Van Oosten 2022) simultaneously accounted for stereotyping and projection, relying on perceived similarity toward the target as a moderator for the inferential strategy selection. Another work (Scaduto and Negri 2024) tried observing which inferential strategy unfolded by inspecting open answers.

Most studies are US-based (71.7 percent). While inflated by the exclusive collection of English-written works,^14^ such a percentage still conveys the US dominance on this topic. Studies have also been conducted in Canada (Bouchard 2022; Kevins and Lee 2023; Ouellet and Tremblay-Antoine 2024), Brazil (Samuels, Mello, and Zucco 2024), the UK (Kevins and Lee 2023; Titelman 2023; Titelman and Lauderdale 2023), Italy (Castelli, Arcuri, and Carraro 2009; Casiraghi, Curini, and Nai 2024; Scaduto and Negri 2024), Belgium (Van Trappen 2023), France (Licata and Méon 2024), Spain (López Ortega 2024), Norway (Arnesen, Duell, and Johannesson 2019), Germany, and the Netherlands (Van Oosten 2022). Only two studies collected evidence in multiple national contexts (Van Oosten 2022; Kevins and Lee 2023), while Bittner (2015) offered comparative evidence in seven countries through secondary data analysis.

With one exception (Bouchard 2022), all the studies follow quantitative methodologies, particularly survey experiments (73.6 percent). Conjoint experiments are often employed in research on A→P (Goggin, Henderson, and Theodoridis 2020; Barber and Pope 2022; Van Oosten 2022; Casiraghi, Curini, and Nai 2024; López Ortega 2024; Myers 2023; Titelman 2023; Titelman and Lauderdale 2023; Ouellet and Tremblay-Antoine 2024). Most works use representative or convenience samples, while Van Trappen (2023) specifically samples party selectors. Some studies were conducted inside a lab (Castelli, Arcuri, and Carraro 2009; Clifford 2014; Coronel and Federmeier 2016; Deichert 2019), while one took place directly in the field during the 2016 Iowa caucuses (Scheffer et al. 2022). Almost all data are cross-sectional, with only two studies (Winter 2010; Busby, Howat, and Daniel Myers 2024) observing PCL over time.

Given the heterogeneity in traits, targets, methods, contexts, and theoretical frameworks employed by these studies, and the changing nature of these associations over time (Busby, Howat, and Daniel Myers 2024), identifying common and contrasting findings is not simple. Some interesting comparisons can nonetheless be discussed. For example, Ahler and Sood (2018) find that people overestimate the number of evangelical Republicans,^15^ and Goggin, Henderson, and Theodoridis (2020) found being evangelical to be associated with a candidate’s Republican partisanship. In contrast, Myers (2023) finds a nonsignificant effect when the inferential targets are Republican partisans. Americans also overestimate the percentage of Black Democrats (Ahler and Sood 2018) and use being Black to infer that someone is not a Republican (but not necessarily to infer Democratic partisanship; Myers 2023). Yet, when the inferential target is a politician, being Black is associated with being more liberal only by white people (Jacobsmeier 2014). Regarding moral traits, Scheffer and colleagues (2022) agree with Clifford’s (2020) finding that Democrats are perceived as more compassionate. Accordingly, Graham and colleagues (2012) and Bruchmann and colleagues (2018) find that liberals are perceived as prioritizing individualizing moral foundations (harm/fairness).

Finally, the categories listed could be crossed to create typologies to further classify these studies. One such typology, arising from crossing the inferential strategy and direction considered, is reported in table 2.

**Table 2.** Classification of the research on PCL according to inferential strategy and direction.

	Inferential strategy		
		Stereotyping	Projection/counter-projection
Direction of the inference	A → P	Research on the use of political stereotypes to detect others’ political traits (49.0 percent)	Research on the projection/counter-projection of political traits toward targets with shared apolitical traits (9.0 percent)
		(among others: Carlson and Settle 2022; Hiaeshutter-Rice et al. 2023; Lee 2021)	(among others: Amira 2018; Kevins and Lee 2023; Lerman and Sadin 2016)
	P → A	Research mapping the apolitical associative network around political traits (38.0 percent)	Research on the projection/counter-projection of apolitical traits to targets with shared political traits (6.0 percent)
		(among others: Ahler and Sood 2018; Busby et al. 2021; Busby, Howat, and Daniel Myers 2024; Rothschild et al. 2019)	(among others: Castelli et al. 2009; Denning and Hodges 2022)

## Research Gaps and Future Directions

As shown in figure 2, the number of published studies on PCL has boomed since 2022. This section discusses the most relevant and fruitful future avenues regarding this research topic.

First, to support the idea that A→P and P→A are both manifestations of underlying associations, therefore reflecting the existence of PCL, observing statistical associations between the propensities to engage in A→P and P→A is crucial. Hiaeshutter-Rice and colleagues (2023) first asked respondents which apolitical traits they associated with parties and then used these answers to observe A→P on a different sample. Yet, we are unaware of studies observing both behaviors from the same subjects, for example, through panel surveys. Moreover, showing that the two facets of PCL share the same moderators may also contribute to arguing for their connection.

Relatedly, another promising line of research concerns investigating factors influencing the propensity to engage in PCL. Studies suggest that PCL is associated with higher ideological polarization (Busby et al. 2021; Claassen et al. 2021), affective polarization (Rothschild et al. 2019; Busby et al. 2021; Claassen et al. 2021; Goovaerts et al. 2024), and social distancing from the political outgroup (Samuels, Mello, and Zucco 2024). Moreover, media exposure (Ahler and Sood 2018; Scaduto and Negri 2024), partisan strength, interest in politics (Ahler and Sood 2018; Carlson and Settle 2022), and conflict avoidance (Carlson and Settle 2022) have also been observed to be associated with one’s propensity to engage in PCL. More research is needed to map the individual factors correlating with PCL. Studies could also observe the situational factors favoring this behavior, such as an environment’s level of political homogeneity, élite polarization, or the proximity to a political election. Finally, future works should also attempt to unpack the causal relationships between PCL and its correlates, particularly polarization (Goovaerts et al. 2024).

While studies abundantly observed PCL performed on people or politicians, the media—the third actor in the political communication arena (McNair 2018)—has been overlooked as a possible inferential target.^16^ Studies could, for example, observe the inferred partisanship of a TV broadcast advertising fast foods and sport cars, or whether a right-wing newspaper is expected to report football news more than a left-wing one. We could observe whether PCL is consequential in selecting the content consumed. For example, an article warning about the health-related risks of a vegan diet may lead people to assume a newspaper leans conservative, affecting the perceived trustworthiness, or the information that a TV series is produced by a liberal TV network may lead to expect more cerebral content or LGBTQIA+ characters, influencing the likelihood of watching it.

There are crucial unaddressed differences between the subcategories of apolitical traits. For example, these can be ascribed or adopted. While sociodemographic characteristics are often ascribed (e.g., ethnicity), one could be considered partially responsible for their psychological traits and fully accountable for their lifestyle preferences. Do adopted traits lead to more confident inferences and engender more serious social consequences? Moreover, traits can be visible (e.g., age, hairstyles) or invisible (e.g., intelligence, music preferences). If PCL is performed on visible traits, before any interaction, its consequences on cross-group contacts may be even more pernicious. Lifestyle traits—so far overlooked by research—are often both adopted and visible. Therefore, we particularly urge future studies to focus on them.

Research has shown that subjective perceptions about the partisan preferences of groups form around sociodemographic traits (e.g., ethnicity, religion), and the subjective feelings toward these groups may shape people’s partisanship (see the “group sentiment model of partisanship,” Kane, Mason, and Wronski 2021; see also “subjective social sorting,” Mason and Wronski 2018). Yet, to our knowledge, no study has tried to observe these effects with groups formed around shared psychological or lifestyle traits.

Most studies on PCL have only considered stereotyping as the inferential strategy. By accounting for perceived similarity, suggested to moderate the selection of inferential strategies (Ames 2004; Denning and Hodges 2022), researchers could glimpse whether stereotyping, projection, or counter-projection is witnessed. In doing this, previous examples (Lerman and Sadin 2016; Van Oosten 2022) should be followed.

Understanding which kind of inference is observed is crucial for crafting interventions tackling the negative consequences of PCL. These interventions should aim at favoring projection rather than stereotyping and counter-projections. Examples are advertising actual similarities between political outgroups (e.g., “Liberals love pizza too”; see Górski, Atkisson, and Hołyst 2023), or favoring awareness of commonalities through cross-group conversations (Hartman et al. 2022).

Filling the contextual gap is crucial to enhance the external validity of current findings. Yet, researchers should be aware of each context’s political and cultural peculiarities. For example, in multiparty systems, partisanship may not be as salient as in the United States. Given cross-national evidence showing that people often interpret social reality through ideological lines (Vegetti and Širinić 2019), ideology might be a more stable political trait for observing PCL in these contexts. Moreover, each country’s unique levels of alignment between political and apolitical traits, cultural norms, sociodemographic compositions, media landscapes, and élites’ behaviors may impact the magnitudes of the effects observed.

When selecting apolitical traits for observing PCL, studies have often relied upon arbitrary choices or studies concerning the alignment. This approach eventually overlooks relevant traits through which people make sense of the political reality. Qualitative and mixed-methods research—currently severely underrepresented—could help provide solid claims about which apolitical traits are likelier to be involved in these inferences. Future studies could employ focus groups to observe shared associations between political and apolitical traits and investigate their origins and the justifications people make for these. For example, Bouchard (2022; see also Knops et al. 2023) showed how participants justified inferring that a Black politician is against Québec’s independence by claiming that immigrants prefer stability, suggesting the mediation of a psychological trait in the path from the observation of a sociodemographic trait to inferring issue preferences.

Doubts remain on whether these inferences are indeed performed in everyday settings or are instead artifacts resulting from the predominant use of surveys to observe them. Field experiments could be valuable for observing the occurrence and consequences of PCL in natural settings. For example, researchers could observe whether older males wearing suits (Deichert 2019) are less likely to be approached by progressive canvassers in the streets, or whether people are less prone to converse with young girls with brightly colored hair during a conservative fundraising event. Moreover, visual stimuli like photographs may be employed to observe if people perform PCL at first sight (Deichert 2019; Hiaeshutter-Rice, Neuner, and Soroka 2023), and recent developments in image generation and recognition algorithms could help design innovative empirical strategies.

## Conclusions

This work introduced a framework for studying political inferences from apolitical cues and apolitical inferences from political cues, two phenomena we subsumed under the concept of politicultural linking (PCL). We departed by rigorously defining PCL and related concepts, such as alignments and associations. Then, we reviewed empirical studies on PCL, highlighting the common elements and categories through which these could be classified: the inferential direction, target, and strategy considered, the apolitical and political traits observed, the national context selected, and the empirical strategy adopted. Through this review, we identified and discussed the gaps in the literature and the promising avenues for future studies. We urged research to investigate the relationship between the two inferential directions, expand its scope to new inferential targets and understudied national contexts, focus on lifestyle preferences as the apolitical trait, account for perceived similarity to observe which inferential strategy unfolds, and supplement survey-based methods with other empirical strategies.

This work is not free from limitations. First, our review, although comprehensive, is limited to the last 15 years. This allowed us to discuss a homogeneous and recent corpus of studies, but it excluded older contributions. Moreover, given the current fragmentation of this literature, we employed a semi-systematic review approach, limiting the strength of our conclusions. Given the recent exponential growth of studies on PCL, we believe it will be possible to conduct systematic reviews on them in the next few years.

Summing up, PCL introduces an innovative way to frame research in political psychology, sociology, and political science dealing with inferences between political and apolitical traits. It allows researchers to consider multiple inferential strategies and both possible directions of the inferences simultaneously. This contribution brought different, hitherto fragmented and dispersed, streams of literature under a common conceptual framework, and points in fruitful directions for future empirical works around PCL, its causes, and its consequences, offering interested scholars an exciting research agenda to pursue in the coming years.

## Supplementary Material

nfaf047_Supplementary_Data

## Data Availability

Replication data and documentation are available at https://doi.org/10.7910/DVN/34ASFV.

## Footnotes

1 The word “politicultural” is mutated by “politicultural sorting” (Rogers 2022), referring to the correlation between political preferences and preferences regarding cultural products/activities. Yet, here we employ a broader meaning of “cultural,” namely everything not fitting into our definition of political (see section 2).

2 This idea relates to the issue-based polarization meta-perceptions discussed by Lees and Cikara (2021), which easily translate to apolitical traits. The belief that the political ingroup is perceived by others through certain apolitical associations might influence one’s own apolitical behavior.

3 We use “traits” as a catchall term to indicate both (static or dynamic) political and apolitical attributes, characteristics, cues, or preferences.

4 The definition was crafted abductively (Timmermans and Tavory 2012): in collecting the studies discussed in section 3, we departed from preexisting ideas on which traits are unequivocally political (e.g., Republican partisanship) and apolitical (e.g., favorite season). After conducting the review, we formulated a definition of political traits fitting all the studies reviewed. While this approach constrains the flexibility of the definition compared with a deductive one, it provides clear boundaries to identify whether an inference is classifiable as PCL.

5 For detailed discussions on how associations might arise, we refer the reader to Deichert (2019), Myers (2020), and Scaduto and Negri (2024).

6 While we did not exclude unpublished works from our review, we recognize our limited ability to retrieve them. The reader should be aware that our considerations might suffer from publication bias (Wagner 2022).

7 For Google Scholar, we stopped when no relevant result was found for five consecutive result pages.

8
https://www.litmaps.com/.

9 We excluded studies about inferences between political and innate physical traits, such as facial features. These studies present homogeneous characteristics distinguishing them from the other studies collected, such as concerning exclusively one inferential direction (A→P), collecting evidence in small-sample laboratory experiments, and not accounting for projection. We refer the interested reader to Carpinella and Johnson (2016) and Lyons and Utych (2022) for reviews. We replicate the categorizations of table 1 for the studies we found within this stream of literature in the Supplementary Material.

10
Deichert (2019) considers both but in separate studies.

11 This subcategory matches Busby and colleagues’ (2021; see also Rothschild et al. 2019) “identity stereotypes,” while the previous matches their “group stereotypes.”

12 See Moral Foundations Theory (Graham, Haidt, and Nosek 2009).

13 Being “antipetista”: against Brazilian Workers’ Party.

14 Moreover, English-speaking countries spend more on scientific research (Man et al. 2004).

15 Especially if they are evangelical themselves (Claassen et al. 2021).

16
Settle (2018) hints at it.
