# Supplementary material for: Politicultural Linking: Inferences Between Political and Apolitical Traits
Source: Public Opin Q. 2025 Nov 1;89(3):915–46. doi: 10.1093/poq/nfaf047 (PMC12695054; doi:10.1093/poq/nfaf047)
Supplement: nfaf047_Supplementary_Data [file nfaf047_supplementary_data.pdf]

Supplementary Material for Politicultural Linking: Inferences between Political and Apolitical Traits

Gaetano Scaduto, PhD Candidate, Department of Sociology and Social Research, University of Milan Bicocca, Milan, Italy, 0000-0002-1368-2077

Corresponding author: Gaetano Scaduto, Sint-Jacobstraat 2, 2000, Antwerpen (BE), gaetano.scaduto@unimib.it

Table of Contents

Classification of Studies Dealing with Political Inferences from Physical Traits 2

References 2

Classification of Studies Dealing with Political Inferences from Physical Traits

Study	Found evidence?	Target of the inference	Political preference	Apolitical trait	Direction of the inference	Mentioned type of inference	National context	Empirical strategy
Carpinella & Johnson, 2013	Partial	Politicians	Partisanship	Other (Physical)	A→P	None	US	Survey experiment + Lab experiment
Herrmann & Shikano, 2016	Partial	Politicians	Ideology	Other (Physical)	A→P	Stereotyping	GE	Lab experiment
Lyons & Utych, 2022	Yes	People	Partisanship	Other (Physical)	A→P	Stereotyping	US	Survey experiment
Olivola et al., 2012	Partial	Politicians	Partisanship	Other (Physical)	A→P	Stereotyping	US	Lab experiment
Petsko & Kteily, 2023	Yes	People	Ideology	Other (Physical)	A→P	Stereotyping	US	Survey experiment
Roberts et al., 2011	Partial	Politicians	Partisanship	Other (physical)	A→P	Stereotyping	UK	Lab experiment
Rule & Ambady, 2010	Yes	People+Politicians	Partisanship	Other (Physical)	A→P	Stereotyping	US	Lab experiment
Wilson & Rule, 2014	Yes	People+Politicians	Partisanship	Other (Physical)	A→P	None	US	Survey experiment

References

Carpinella, C. M., & Johnson, K. L. (2013). Appearance-based politics: Sex-typed facial cues communicate political party affiliation. *Journal of Experimental Social Psychology*, *49*(1), 156–160. https://doi.org/10.1016/j.jesp.2012.08.009

Herrmann, M., & Shikano, S. (2016). Attractiveness and Facial Competence Bias Face-Based Inferences of Candidate Ideology. *Political Psychology*, *37*(3), 401–417. https://doi.org/10.1111/pops.12256

Lyons, J., & Utych, S. M. (2022). Partisan discrimination without explicit partisan cues. *Journal of Social and Political Psychology*, *10*(1), 288–305. https://doi.org/10.5964/jspp.6491

Olivola, C. Y., Sussman, A. B., Tsetsos, K., Kang, O. E., & Todorov, A. (2012). Republicans Prefer Republican-Looking Leaders: Political Facial Stereotypes Predict Candidate Electoral Success Among Right-Leaning Voters. *Social Psychological and Personality Science*, *3*(5), 605–613. https://doi.org/10.1177/1948550611432770

Petsko, C. D., & Kteily, N. S. (2023). Political (Meta-)Dehumanization in Mental Representations: Divergent Emphases in the Minds of Liberals Versus Conservatives. *Personality and Social Psychology Bulletin*, 01461672231180971. https://doi.org/10.1177/01461672231180971

Roberts, T., Griffin, H., McOwan, P. W., & Johnston, A. (2011). Judging Political Affiliation from Faces of UK MPs. *Perception*, *40*(8), 949–952. https://doi.org/10.1068/p6985

Rule, N. O., & Ambady, N. (2010). Democrats and Republicans Can Be Differentiated from Their Faces. *PLoS ONE*, *5*(1), e8733. https://doi.org/10.1371/journal.pone.0008733

Wilson, J. P., & Rule, N. O. (2014). Perceptions of Others' Political Affiliation Are Moderated by Individual Perceivers' Own Political Attitudes. *PLoS ONE*, *9*(4), e95431. https://doi.org/10.1371/journal.pone.0095431
